# Overexpression of an auxin receptor *OsAFB6* significantly enhanced grain yield by increasing cytokinin and decreasing auxin concentrations in rice panicle

**DOI:** 10.1038/s41598-018-32450-x

**Published:** 2018-09-19

**Authors:** Qin He, Lin Yang, Wei Hu, Jia Zhang, Yongzhong Xing

**Affiliations:** National Key Laboratory of Crop Genetic Improvement and National Center of Plant Gene Research (Wuhan), Huazhong Agricultural University, Wuhan, China

## Abstract

Auxin plays critical roles in many developmental processes of plants. The auxin signaling pathway is a series of plant responses to auxin stimuli. However, the functions of many genes in this pathway are still obscure. As auxin receptors, *TIR/AFB* family genes encode F-Box proteins that directly bind auxin and then transduce the stimulus through the signaling pathway. In this paper, we generated an overexpression line of *Auxin-signaling F-Box 6* (*OsAFB6*) in rice, which largely delayed heading, greatly increased spikelets per panicle and primary branch number and ultimately enhanced grain yield by 50%. *OsAFB6* is preferentially expressed in young tissues with stronger meristem activities and suppresses flowering by upregulating *OsRR1* and downregulating *Ehd1* expression levels. Overexpression of *OsAFB6* delayed heading, increased cytokinin (CK) by suppressing the expression level of *Gn1a* and simultaneously decreased the IAA concentration in the young panicle, which promoted inflorescence meristem development and resulted in large panicles with more spikelets per panicle, primary branches and increased grain yield. It would be a beneficial strategy to generate lines with varied expression levels of *OsAFB6* to breed high-yielding cultivars for specific regions that can fully utilize the local sunlight and temperature resources.

## Introduction

Auxin regulates many aspects of plant growth and development, including embryogenesis^1^, the architecture of the root system^2^, gravitropism^3^, phototropism^4^, initiation and radial positioning of plant lateral organs and cell elongation^5,6^. Plants sense auxin by receptors and then transduce its signal to elicit a series of responses. The intracellular receptors of auxin are the Transport Inhibitor Response 1/Auxin-signaling F-Box (*TIR1/AFB*) gene family^7–9^. *TIR1/AFB* genes encode F-box proteins that have an amino-terminal F-box motif for mediating interactions with SKP1 and form part of the SCF (Skp-Cullin-F-Box containing protein) complex, a carboxy-terminal protein-protein interaction domain for targeting specific substrates Auxin/Indole-3-Acetic Acid (*Aux/IAA*), and a leucine-rich repeat (LRR) domain^10^. *TIR1* is the first auxin receptor identified in this family^7,8^. Other *AFB* members have also been demonstrated to work as auxin receptors^11,12^ but with diverse functions. For example, *OsTIR1* and *OsAFB2* are targets of microRNAs. Overexpression of *OsmiR393* downregulates both genes and results in more tillers, early flowering and less tolerance to salt and drought stress^13^. Both *OsTIR1* and *OsAFB2* regulate the auxin response but differ in their relative contributions to seedling development^9^. The *AFB*s contribute to auxin response, with diverse intensities of interactions with *Aux/IAA*s^9^. *AFB4* and *AFB5* in *Arabidopsis* have been confirmed to respond specifically to synthetic auxin picloram. Mutants for both genes are resistant to picloram with no elongation of hypocotyls and are suitable for the analysis of resistance to auxin-like herbicides^12^.

The TIR1/AFB family members mediate the auxin signaling pathway, which is centered on ubiquitin-dependent SCF^TIR1/AFBs^-Aux/IAAs-ARFs flow^14^. With low auxin concentration, Aux/IAAs repress the transcriptional activities of auxin response factor genes (*ARF*s) by recruiting the corepressor TOPLESS. As auxin levels increase, the auxin receptor TIR1/AFB, a subunit of an E3 ubiquitin-protein ligase SCF complex, binds to auxin directly and recognizes the specific target Aux/IAAs, which are then degraded through ubiquitination^15^. Subsequently, the repressed ARFs are released and function as either activators or repressors to regulate downstream genes in the auxin signaling pathway^16^. There are six TIR1/AFBs in rice^9^ that degrade 31 Aux/IAAs^17^, which in turn repress 25 ARFs^18^, resulting in the regulation of other downstream genes. The number of possible combinations of TIR/AFB-Aux/IAA-ARF is large and results in a complex auxin-dependent regulation network. The functions of some combinations have been well characterized^19,20^.

Similar to auxin, cytokinin (CK) is another important plant hormone regulating many aspects of plant growth^21,22^. CK plays positive roles in the regulation of shoot apical meristem (SAM) activity^23^. The *LOG* (*LONELY GUY*) gene encodes a novel cytokinin-activating enzyme that works in the final step of bioactive cytokinin synthesis and shows specific expression at the top of shoot meristems where stem cells reside and CK accumulates^24^. Auxin and CK act either synergistically or antagonistically to play roles in several significant developmental progresses^25^. Maintenance of the optimum cellular concentration of auxin and CK is rather important and can be regulated in multiple processes, such as biosynthesis, transport, perception, signaling and degradation^26^.

Auxin and cytokinin can regulate grain yield. For example, *PIN*s, encoding auxin efflux transporters, with the pin-formed mutant phenotype, are regulated by an auxin-dependent Aux/IAA-ARF signaling pathway^27^. A recent study demonstrated that the auxin transporter *PIN5b* gene could modulate rice plant architecture and yield^28^. CK also makes a great contribution to regulating spikelets per panicle, which directly associates with grain yield^29^.

Rice heading date is a growth-adaptive trait that is related to grain yield and is controlled by multiple genes. A gene family comprising 41 members encoding CCT (CO, CO-LIKE and TOC1) domain proteins plays important roles in regulating heading date. The major heading date genes, including *Hd1*^30^, *Ghd7* and *Ghd7.1*, belong to the CCT family^31,32^. In addition to these three genes, nine additional CCT genes were confirmed to control flowering in rice^33^. *OsHAP*, another flowering-related gene family containing *Ghd8*^34,35^, is also a pleiotropic gene regulating heading date, plant height and spikelets per panicle, similar to *Ghd7*.

In the process of overexpressing *CCT05*, we generated a late-flowering line. However, this phenotype change was not associated with *CCT05* overexpression. In this study, we confirmed that overexpression of the auxin signaling F-box gene *OsAFB6* resulted in late flowering. It was also noted that plants with *OsAFB6* overexpression exhibited more primary branches, more spikelets per panicle, and a higher grain yield. We demonstrated that *OsAFB6* regulated key genes involved in flowering pathways and panicle formation-related hormone pathways.

## Results

### Cloning and functional characterization of *OsAFB6*

**Figure 1:**
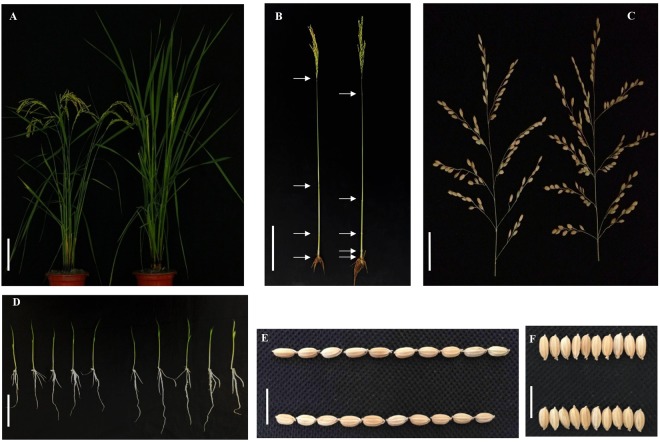
Performance of OX*OsAFB6* mutant plants.The whole plants. (**A**), main culms (**B**), panicles (**C**) and seedlings (**D**) for the control plant (left) and *OsAFB6* overexpression mutant (right); the grain length (**E**) and grain width (**F**) for the control plants (up) and *OsAFB6* overexpression plants (bottom). Scale bars, 20 cm **(A**) and (**B**), 5 cm (**C**) and (**D**), and 1 cm (**E**) and (**F**).

To test the function of *CCT05* (LOC_Os02g08150), *CCT05* RNA interference plants and *CCT05*-overexpressing plants were generated. Transcript levels of *CCT05* in the RNAi plants were knocked down to 10–30%, but no differences in phenotypes were observed between the positive and control plants^33^. Interestingly, among the three *CCT05* overexpression lines, only one exhibited variability in heading date, with a segregation ratio of 3:1; late flowering was dominant over early flowering (Fig. 1A). However, the transcript level of the *CCT05* gene in this family was not detectibly overexpressed (Fig. S1). Therefore, we considered the late-flowering transgenic plant as a new mutant, and the heading date variation was not caused by *CCT05* overexpression but by other gene alterations during the transformation event.

Southern blot hybridization confirmed a single copy insertion in the mutant (Fig. S2A), consistent with the single gene segregation ratio of 3:1 in the T1 progeny. Thermal asymmetric interlaced PCR (TAIL-PCR) analysis showed that the vector was inserted at 0.56kb upstream of the gene LOC_03g08850 (Fig. S2B), the auxin-signaling F-Box (AFB) gene *OsAFB6*. To confirm that *OsAFB6* was the only gene affected by the insertion, the expression levels of all the genes near the insertion site were investigated (Fig. S2C–E). While adjacent genes showed comparable transcript levels to the control plants, *OsAFB6* was the only gene that had significantly increased expression levels in the mutant at the seedling stage; moreover, the increased amount under SD conditions (5 to 20-fold, Fig. S2G) was significantly lower than under LD conditions (15 to 35-fold, Fig. S2E). In addition, *OsAFB6* expression exhibited a circadian rhythmic pattern, with high expression during daytime that decreased dramatically at night (Fig. S2F–H). These results highly suggest that *OsAFB6* is a strong candidate gene for the mutant phenotype.

To further test this idea, an F_2_ population of 40 plants was developed by selfing the hybrid of the mutant and wild type (ZH11). Higher *OsAFB6* expressions were detected in all 28 late-flowering plants, whereas levels similar to those in the control plants were detected in the 12 early-flowering plants (Fig. S3). This cosegregation test indicated that *OsAFB6* was the gene responsible for late heading in the mutant. Hence, the mutant, hereafter named OX*OsAFB6*, was the result of *OsAFB6* overexpression. The seeds of OX*OsAFB6* were deposited in the China Center for Type Culture Collection (CCTCC) with accession number P201709.

### Overexpression of *OsAFB6* can delay heading and increase grain yield

In addition to the heading date variation, we found that the mutant was also photoperiod-sensitive, as flowering was delayed by ~22 days under LD conditions and by ~12 days under SD conditions (Table 1). In addition, the mutant had fewer tillers (Fig. 1A), one more internode in the main culm, longer panicles with more primary and secondary branches in total, more spikelets per panicle (Fig. 1B,C and Table 1), and longer roots at the seedling stage (Fig. 1D). Although the grain size was smaller (Fig. 1E,F), the grain yield per plant was significantly increased by approximately 50% under both LD and SD conditions (Table 1).

**Table 1:** Comparison of yield related phenotypes between *OsAFB6* overexpression mutants and its control plants under LD and SD conditions.

Conditions	Traits	Control plants	OX*OsAFB6* mutants	*P* value
LD (Wuhan)	Heading date (d)	73.6 ± 0.6	95.6 ± 1.1	1.1E-10
	Spikelets per panicle	125.6 ± 9.3	175.3 ± 14.8	5.7E-05
	Grain yield per plant (g)	27.0 ± 5.1	44.3 ± 8.0	9.9E-03
	panicle length (cm)	20.9 ± 0.9	24.7 ± 1.5	5.1E-10
	grain length (mm)	6.8 ± 7E-2	6.5 ± 0.1	7.0E-05
	grain width (mm)	2.9 ± 5E-2	2.8 ± 3E-2	2.9E-03
	kilo-grain weight (g)	23.2 ± 1.4	20.5 ± 1.0	9.9E-04
	primary branch number	8.8 ± 0.9	10.3 ± 1.0	7.8E-04
	secondary branch number	25.1 ± 3.9	34.5 ± 6.5	2.4E-04
SD (Hainan)	Heading date (d)	61.6 ± 0.6	73.8 ± 3.1	1.3E-05
	Spikelets per panicle	61.9 ± 3.9	98.4 ± 13.0	8.7E-04
	Grain yield per plant (g)	18.1 ± 6.5	28.1 ± 12	1.7E-03
	panicle length (cm)	17.7 ± 1.3	20 ± 0.6	9.7E-03
	grain length (mm)	6.7 ± 0.1	6.6 ± 0.1	3.7E-02
	grain width (mm)	2.8 ± 0.1	2.7 ± 0.1	2.5E-02
	kilo-grain weight (g)	23.5 ± 0.5	22.2 ± 0.9	5.9E-03
	primary branch number	7.3 ± 0.5	8.6 ± 0.8	6.3E-04
	secondary branch number	13.4 ± 2.7	22.7 ± 4.6	1.8E-04
	Root length (cm)	7.2 ± 1.1	8.6 ± 1.6	1.0E-02

Plants for root length measurement were cultivated on MS media for 15 days under short-day condition. Data was represented by mean ± standard deviation.

To further verify whether the various phenotypes were caused by the upregulation of *OsAFB6*, *OsAFB6* was overexpressed in ZH11 under the control of the 35S promoter. We generated 18 transgenic positive plants. T1 lines from all 18 plants segregated with early- and late-flowering phenotypes in both Hainan (winter, 2015) and Wuhan (summer, 2016). *OsAFB6* was overexpressed in all late-flowering phenotype plants but not in early-flowering plants.

In addition, we took T2 lines from four single-copy inserted transgenic T0 individuals with different expression levels as examples. Line 1 (*OsAFB6* overexpressed approximately 9-fold) delayed flowering by approximately 5 days under SD and 7 days under LD, with an increase in spikelets per panicle and yield but no significant changes in primary branch number. Line 2 (*OsAFB6* overexpressed approximately 19-fold) showed a large delay in heading date (17 days under SD and 19 days under LD) with increased primary branch number, spikelets per panicle and yield; lines 3 and 4 (overexpressed approximately 40 and 45-fold) flowered even later (18 and 19 days under SD and 25 and 26 days under LD, respectively) but with comparable or slightly decreased primary branch number, spikelets per panicle and yield (Table 2). These findings confirmed that the overexpression of *OsAFB6* would result in a later heading date and increased grain yield.

**Table 2:** Performance of *OsAFB6* overexpression lines under LD and SD conditions.

Conditions	Genotype	Heading date (d)	primary branch number	Spikelets per panicle	Grain yield per plant (g)
LD (Wuhan)	Control	76.3 ± 2.2	9.8 ± 0.8	142.5 ± 9.7	22.7 ± 4.7
	Line 1	83.7 ± 3.2**	10.3 ± 1.0	178.3 ± 10.5**	33.4 ± 5.5**
	Line 2	95.3 ± 3.6**	11.8 ± 0.6*	183.6 ± 10.9**	37.9 ± 6.2**
	Line 3	101.4 ± 2.7**	10.2 ± 0.8	148.5 ± 11.7	19.8 ± 3.2*
	Line 4	102.7 ± 3.6**	9.5 ± 0.7	135.7 ± 8.8**	18.9 ± 3.7*
SD (Hainan)	Control	70.6 ± 2.9	8.7 ± 0.6	95.3 ± 1.8	16.4 ± 4.3
	Line 1	75.6 ± 2.9*	9.2 ± 1.0	111.7 ± 11.4**	21.3 ± 6.8**
	Line 2	87.0 ± 3.0**	9.9 ± 0.5*	118.7 ± 10.1**	24.6 ± 6.4**
	Line 3	88.8 ± 1.6**	8.4 ± 0.8	95.1 ± 13.8	14.2 ± 5.6
	Line 4	90.0 ± 3.4**	7.6 ± 0.9	81.6 ± 9.5**	13.9 ± 3.7*

*, ** Significant difference at 5% and 1% level as compared to control lines. Data was represented by mean ± standard deviation.

However, the dsRNA-mediated gene silencing transgenic plants and CRISPR plants of *OsAFB6* in the ZH11 background showed no phenotypic changes (Fig. S4). It is likely that gene redundancy exists among six members in the TIR1/AFB family.

### Expression of *OsAFB6* in the meristem might be associated with increased primary branch number in the OX*OsAFB6* mutant

Sequence analysis showed that *OsAFB6* covered a 2020-bp region of the genome, consisting of three exons and two introns, with a coding sequence of 1812 bp encoding a protein of 603 amino acids. One F-box domain located in its N-terminal region, described as a recognizer for ubiquitination targets, and two AMN1 domains containing many LRR repeats located in the middle and C terminus of the *OsAFB6* protein (Fig. S5). Many cis-acting elements were identified in the promoter (Table S1), including common elements related to transcription activity, elements that respond to light, auxin and other hormones such as ABA and GA, elements involved in the stress response and others.

**Figure 2:**
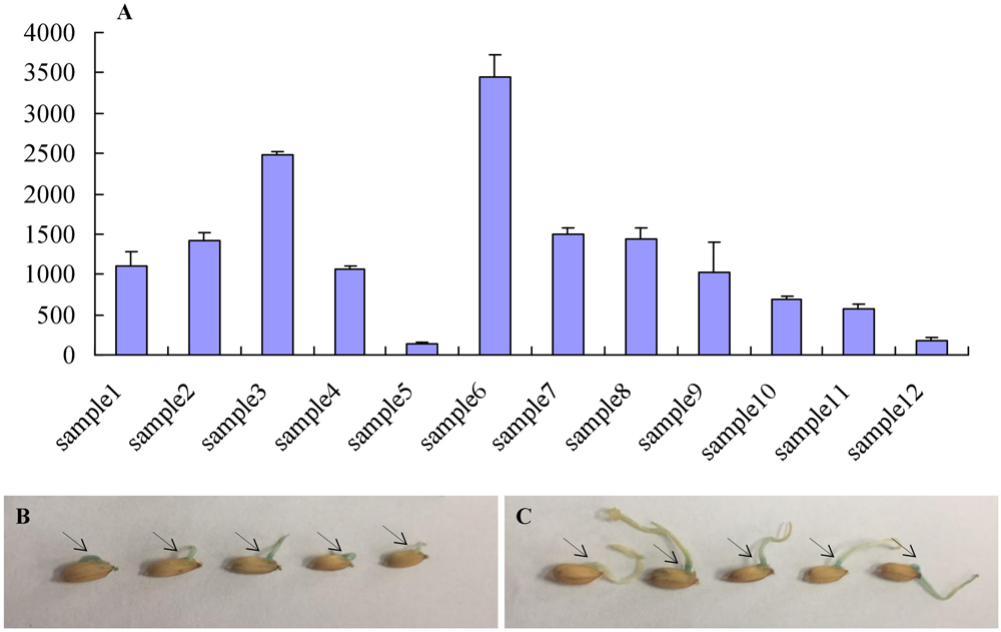
Expression pattern of *OsAFB*6. (**A**) Expression of *OsAFB6* in different tissues and different stages (data from http://crep.ncpgr.cn/crep-cgi/home.pl). Sample1: plumule; sample2: stigma; sample3: ovary; sample4: 7-day-old seedling; sample5: mature leaf; sample6: SAM sample7: young inflorescence (up to 3 cm); sample8: inflorescence (3–5 cm); sample9: inflorescence (5–10 cm); sample10: inflorescence (10–15 cm); sample11: inflorescence (15–22 cm); sample12: inflorescence (22–30 cm). (**B**,**C**) Pictures of the *OsAFB6* promoter-driven GUS transgenic material: (**B**) one day after germination and (**C**) two days after germination. These materials were soaked in GUS reagent for another 24 hours.

The expression profile of *OsAFB6* was extracted from online microarray data (http://crep.ncpgr.cn/crep-cgi/home.pl). *OsAFB6* was highly expressed in tissues such as plumule, shoot apical meristem, young inflorescence and reproductive organs (stigma and ovary) compared to mature leaves and seedlings (Fig. 2A). The *OsAFB6* gene expression level gradually decreased with the growth of the inflorescence. Samples of ovary, inflorescences and stigma were collected from exclusive organs. However, for the young plumule, the samples are collected from the whole plumule (from basal to tip). To test whether the expression of *OsAFB6* probably exhibited gradient changes in plumula, a construct with the GUS gene driven by the 2-kb *OsAFB6* native promoter was introduced into the wild-type plant ZH11. GUS staining showed that the site near the embryo where the plumule came out displayed a stronger signal than the other parts, showing an expression gradient that decreased from the basal part to the tip (Fig. 2B,C). All these results illustrated that from germination to the heading stage, *OsAFB6* is preferentially expressed in tissues where meristem cells with more activities are located.

**Figure 3:**
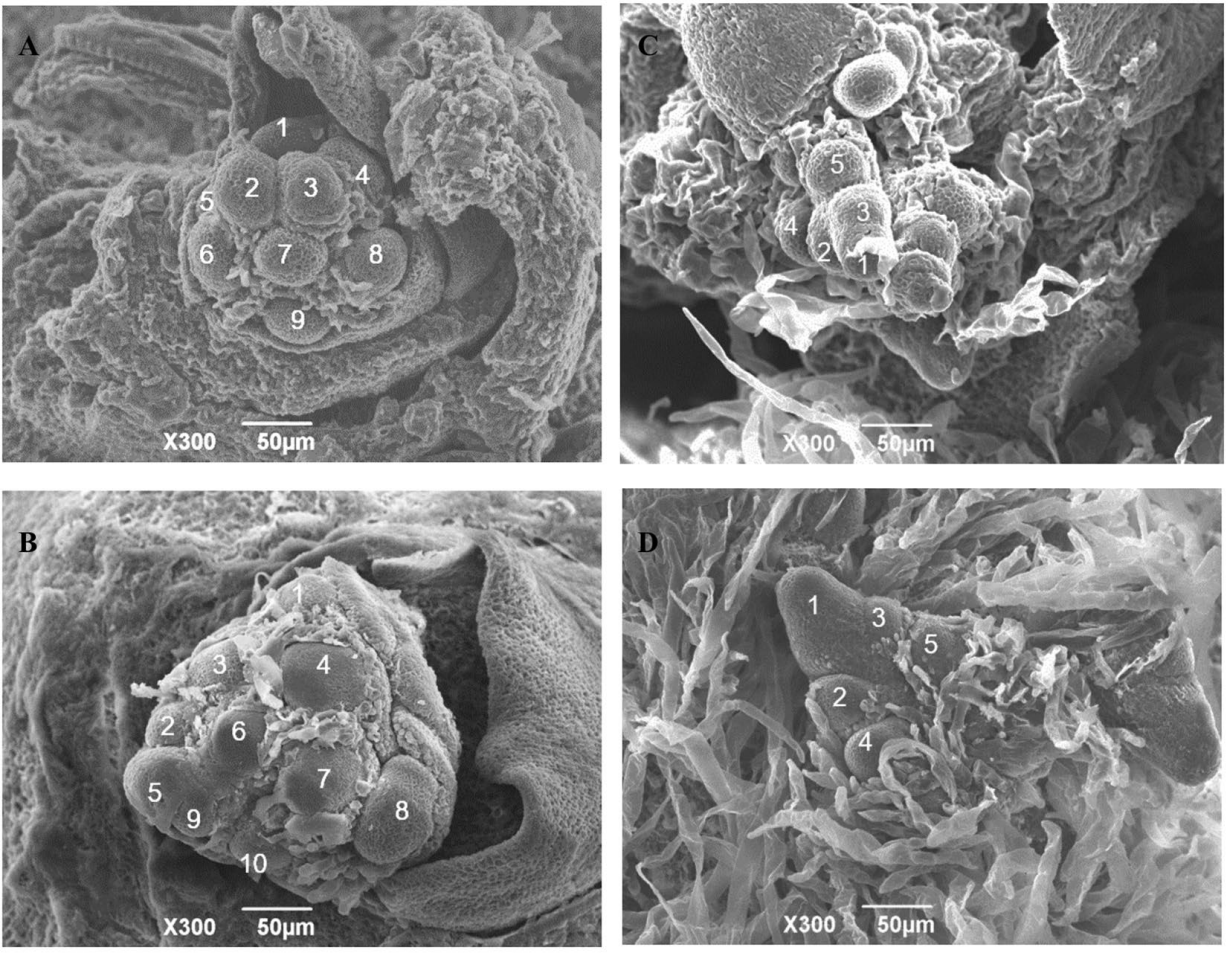
Scanning Electronic Microscope pictures for branch primordia of the OX*OsAFB6* mutants and the control plants. Primary branch primordia of the control plant (**A**) and OX*OsAFB6* mutant (**B**), secondary branch primordia of the control plant (**C**) and (**D**) OX*OsAFB6* mutant. The numbers on the picture indicate the number of branch primordium.

In addition, we performed scanning electron microscopy to identify any cellular changes in tissues with high *OsAFB6* expression. The primary and secondary branches of the young panicles before bract hair initiation were observed in *OXOsAFB6* mutants. The primary branch number was 8.8 in control plants and 10.3 in *OXOsAFB6* mutants, whereas the secondary branch numbers on each primary branch were comparable (approximately 5; Fig. 3).

### *OsAFB6* increased the CK concentration and decreased the IAA concentration in young panicles

**Figure 4:**
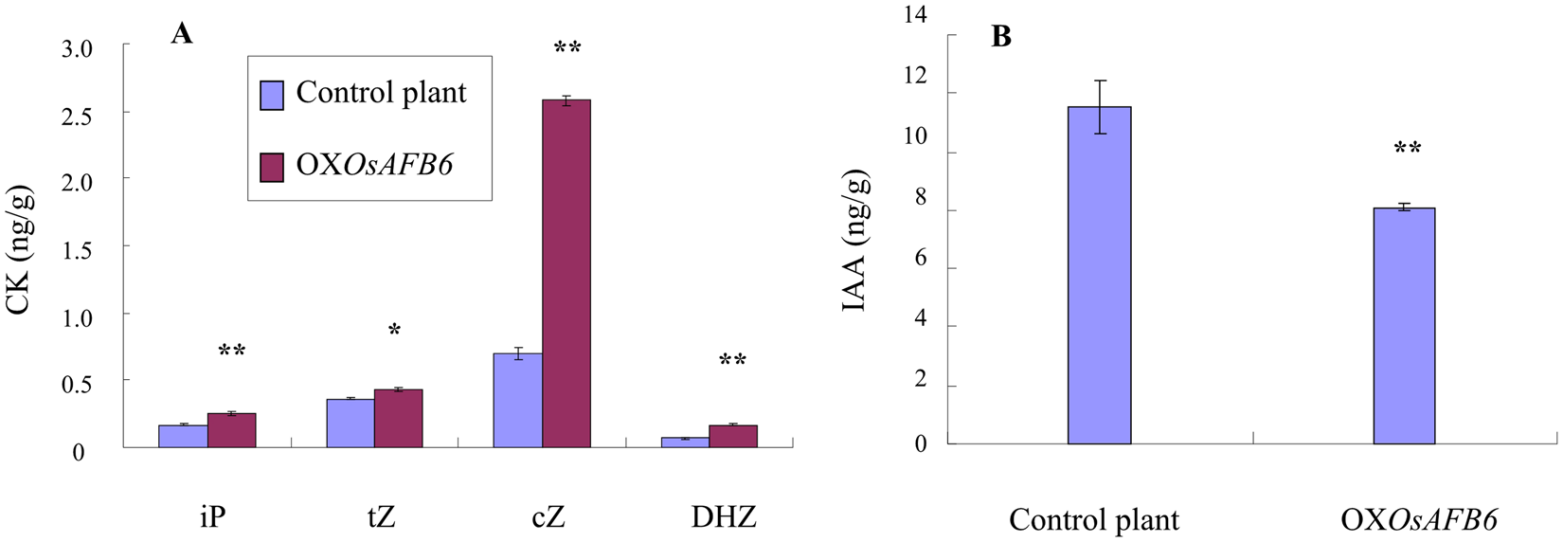
Cytokinin and IAA concentration of young panicles. iP: N6-(Δ2-isopentenyl) adenine. tZ: trans-zeatin. cZ: cis-zeatin. DHZ: Dihydrozeatin. There were four active forms of CK. OX*OsAFB6* and control plants represent the *OsAFB6* overexpression mutant and the Zhonghua 11 plant as the negative control, respectively. Error bars, standard deviation. *, ** Indicates significance at the level of P < 0.05 and 0.01, respectively.

To uncover the function of *OsAFB6* in the hormone signaling pathway, we measured endogenous cytokinin and IAA concentrations in young panicles of the OX*OsAFB6* mutants and the control plants. N6-(Δ2-isopentenyl) adenine (iP), trans-zeatin (tZ), cis-zeatin (cZ), and dihydrozeatin (DHZ) were four active CK forms^36^. Concentrations of all four active CK forms were significantly higher in the *OXOsAFB6* mutant than in the wild type (Fig. 4A), especially cZ with more than a three times increase. However, the IAA concentration was significantly lower in the *OXOsAFB6* mutant plants (*OXOsAFB6* mutant plants: 8.1 ± 0.1 ng/g, control plants: 11.5 ± 0.9 ng/g) (Fig. 4B). In summary, the overexpression mutant of *OsAFB6* increased the CK concentration and decreased the IAA concentration in young panicles.

### *OsAFB6* delays heading date by repressing the key flowering gene *Ehd1*

**Figure 5:**
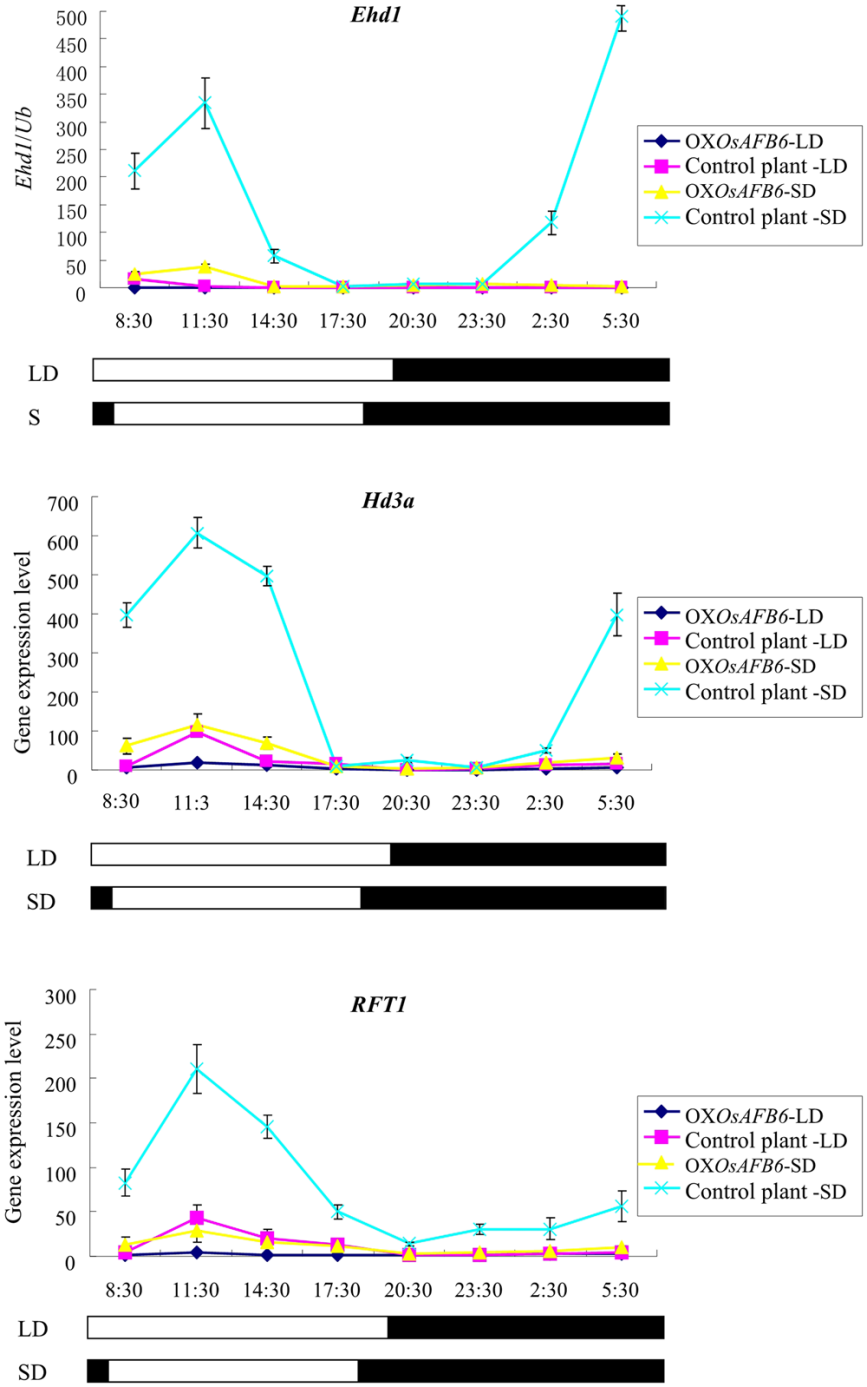
Expression levels of key flowering genes from the photoperiod flowering pathway in the OX*OsAFB6* mutant and control plant under LD and SD conditions. OX*OsAFB6*-LD and control plant-LD represent the genotypes of *OsAFB6* for the expression mutant and the negative the control plant, respectively, under long-day conditions. OX*OsAFB*-SD and control plant-SD represent the genotypes under short day conditions. The white bars indicate the light period, and the black bars indicate the dark period. The numbers below the x-axis indicate hours of the days. Error bars, standard deviation.

As shown above, the mutant OX*OsAFB6* significantly delayed the heading date and showed strong photoperiod sensitivity (Table 1). To elucidate the associated mechanism, seven key genes involved in the photoperiod flowering pathway were transcriptionally examined by qRT-PCR under both LD and SD conditions. Expression level analysis showed that *Ehd1*, as well as the florigen genes *Hd3a* and *RFT1*, was greatly suppressed in OX*OsAFB6* plants (Fig. 5). The expression levels of other genes, such as *OsGI*, *Ghd7*, *Ghd7.1* and *Hd1*, were comparable to those of the control plants (Fig. S6). Hence, *OsAFB6* overexpression dramatically decreased the expression level of florigen genes by repressing *Ehd1*, which ultimately resulted in late flowering.

### Global regulation by *OsAFB6* involved in flowering and hormone pathways

An RNA-seq experiment was also launched to define the global regulation of *OsAFB6* in the *OXOsAFB6* mutant. A total of 287 differentially expressed genes were identified in young panicles. Among these genes, 58 were upregulated, while the remaining 229 were downregulated (Fig. S7; Excel named RNA sequencing result). GO analysis showed that the up- or down-regulated genes were involved in photosystem, which suggests they might be sensitive to light; in flowering pathway, and in hormone pathways (including auxin and other hormones sensitive, biosynthetic, catabolic processes and signaling pathways). For instance, two flowering-related genes, *OsMADS34/PAP2* (LOC_Os03g54170) and *OsHAP2F* (LOC_Os12g42400), were upregulated, and *OsHAP3F* (LOC_Os07g41580) was downregulated. Five genes in auxin signaling, degradation and transport pathways were downregulated, including one of the *Aux/IAA* family genes, *OsIAA27* (LOC_Os11g11410); the auxin-rapid response gene *OsGH3.3/OsJAR2* (LOC_Os01g12160), which also functioned as a light and JA-signaling gene; the dioxygenase for auxin oxidation gene *DAO* (LOC_Os04g39980); a gene that promoted auxin polar transport, *HOX1* (LOC_Os10g41230); and an auxin efflux transporter gene, *OsPIN5b* (LOC_Os08g41720). Genes in other hormone pathways, such as the cytokinin-signaling gene *OsRR11* (LOC_Os02g42060) and *WOX3* (LOC_Os05g02730), which is a gene that might integrate auxin and cytokinin signaling pathways, were upregulated. Four genes in cytokinin pathways were downregulated: cytokinin dehydrogenase *CKX5* (LOC_Os01g56810); *D*14 (LOC_Os03g10620), which functions in the strigolactone (SL) signaling pathway; *OsNAC2* (LOC_Os04g38720), which participates in GA signaling; and *RAF1* (LOC_Os10g30870), which is responsive to ethylene. These data suggest that *OsAFB6* plays an important role in the flowering pathway, the auxin signaling pathway and crosstalk with other hormone signaling pathways.

**Figure 6:**
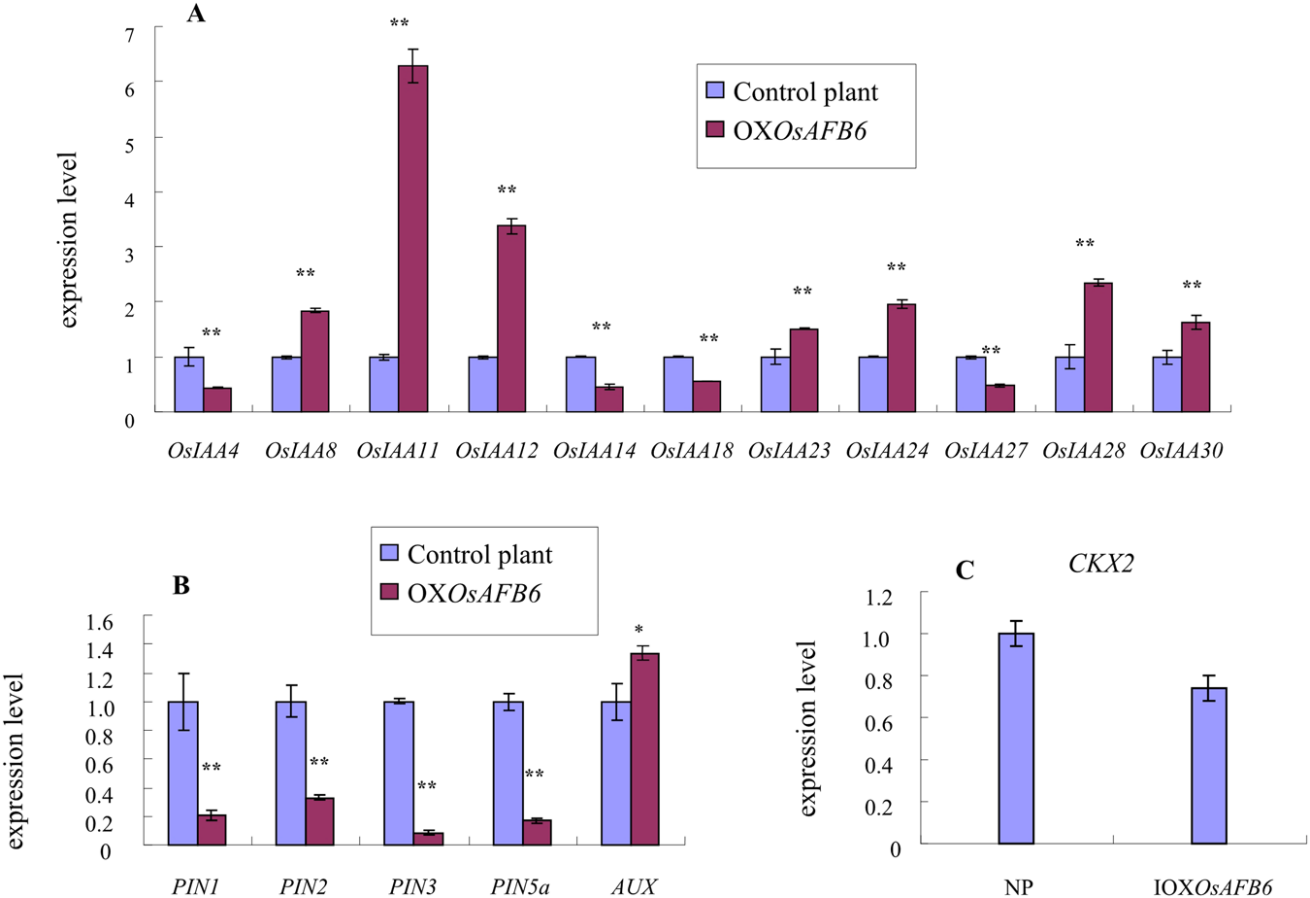
Differentially expressed genes in young panicles by real-time PCR. (**A**) The expression level of *OsIAA/AUX* family genes. (**B**) The expression level of auxin transporter genes. (**C**) CKX2 expression level. OX*OsAFB6* and control plants represent the *OsAFB6* overexpression mutant and Zhonghua 11 plant as the negative control, respectively. The expression levels of each gene in the control plants were marked as 1. Error bars, standard deviation. *, ** Indicates significance at the level of P < 0.05 and 0.01, respectively.

Further experiments were carried out to confirm the expression levels of genes involved in signaling pathways identified through RNA-seq. In rice, there are 31 genes encoding Aux/IAAs, which are short-lived nuclear proteins, the substrates of the SCF complex^17^. *OsIAA27*, a member of the *AUX/IAA* family, was downregulated in the OX*OsAFB6* mutant RNA-seq results. Thus, a total of 31 genes in the family were analyzed by qRT-PCR. Four of them, *OsIAA4*, *14*, 18 and 27, were downregulated, whereas *OsIAA8*, 11, 12, 23, 24, 28 and 30 were upregulated in the mutant (Fig. 6A). An auxin efflux carrier-like gene *OsPIN5b* (LOC_Os08g41720) was downregulated in the *OXOsAFB6* mutant RNA-seq results. Therefore, the expression of the other 5 auxin transporter genes, including the influx transporter gene *AUX1* and efflux transporter genes *PIN1*, 2, 3, and *5a*, was also examined (Fig. 6B). The influx transporter gene *AUX1* was upregulated, while all the efflux transporter genes were downregulated. In addition, *CKX5*, a cytokinin dehydrogenase from the CKX gene family, was found to be downregulated in the *OXOsAFB6* mutant RNA-seq results. Interestingly, we found that another well-known gene in this family, *CKX2* (*Gn1a*), which regulates rice grain production^29^, was also downregulated in the mutant (Fig. 6C). Taken together, our RNA-seq and further experiments show that *OsAFB6* plays a critical role in global flowering and hormone regulatory systems and contributes to the phenotype change of the *OXOsAFB6* mutant in late flowering and grain yield increases.

## Discussion

### *OsAFB6* overexpression delays heading date by repressing the expression of *Ehd1*

Rice is a short-day plant that flowers early under short-day conditions. The *OsAFB6* overexpression mutant delayed heading under LD and SD conditions but with different effects. This character of *OsAFB6* is unique among known flowering genes. *Hd1* promotes flowering under SD conditions but delays flowering under LD conditions^30^. *Ghd7*, *Ghd8, Ghd7.1* and *OsMADS56* are specific repressors under LD conditions^31,32,34,37^. *CO3* negatively regulates flowering under SD conditions^38^. *COL4*, *COL10*, *COL13* and *OsPhyB* are constitutive repressors under both SD and LD conditions^39–42^, while *OsMADS50* can promote flowering under both conditions^43^. *Ehd1*, a well-known flowering gene, is highly homologous to the B-type response regulator (RR) family genes in the CK signaling pathway^44^. A recent study revealed that homodimerization of *Ehd1* was crucial for its activity, which could be inhibited by OsRR1, an A-type RR member. OsRR1 physically interacted with *Ehd1* to form a heterodimer, which was an inactive complex for regulating flowering^45^. In OX*OsAFB6*, *OsRR1* was upregulated, and more OsRR1 proteins were potentially synthesized. With a lower *Ehd1* expression level, more *Ehd1* was involved in forming the inactive heterodimer OsRR1-*Ehd1*; this greatly weakened *Ehd1* flowering promotion. *OsAFB6* was a light-responsive gene harboring light response cis-elements in the promoter region, which probably explained the fact that its transcription could be induced rapidly after moving from dark to light conditions and decreased dramatically under the inverse conditions (Fig. S2F–H), as well as the fact that the expression levels of *OsAFB6* in the OX*OsAFB6* mutant were 5–20-fold and 15–35-fold higher compared with the control plants under SD and LD conditions, respectively. Therefore, the flowering difference was dependent on the *OsAFB6* expression level, which was regulated by photoperiod.

### *OsAFB6* increased grain yield through CK pathways

The plant hormone cytokinin is another key regulator of growth and development in plants, such as the formation of SAM^23^. The CK concentration remained balanced by synthesis and metabolism. The *WOX3* gene was reported to downregulate the expression level of the *YAB3* gene, which encodes a repressor of the *KNOX* genes *OSH1* and *OSH3*^46^. KNOX proteins function as positive regulators of CK production through induction of the CK biosynthesis enzyme^47^. In this study, overexpression of *OsAFB6* upregulated the *WOX3* gene, which resulted in more CK by alleviating the repression of the CK biosynthesis enzyme by the *YAB3* gene. In contrast, *Gn1a* (*OsCKX2*), which encodes cytokinin oxidase/dehydrogenase, an enzyme that degrades the phytohormone cytokinin^29^, was confirmed to be downregulated in the young panicle of the OX*OsAFB6* mutant. This would prevent the degradation of CK and result in CK accumulation, which was reported to regulate rice branch and flower numbers^48^. Therefore, an increased CK level was expected in OX*OsAFB6*. IAA repressed CK biosynthesis^49^ and inhibited apical meristem outgrowth^50^. In fact, the increased endogenous active CK concentration in the mutant OX*OsAFB6* agreed with the expression changes of CK-related genes. Taken together, higher CK and lower IAA concentrations in the panicle promoted inflorescence meristem development and resulted in large panicles in the OX*OsAFB6* mutant. In addition, the differentiation of both the sieve tubes and the vessels of vascular tissues was induced by auxin and CK^51^, which was also in accordance with the observation that there were more vascular bundles in OX*OsAFB6*. The advanced vascular system enhanced assimilate transport capability and ensured a comparable seed setting rate. Finally, the grain yield increased by 50% in the OX*OsAFB6* line compared to the control plants (Table 1).

### Potential of *OsAFB6* for high-yield breeding in different regions

There is a large planting zone for rice worldwide; some regions have a long day length and some have a neutral day length or short day length during the rice-growing season. Therefore, the varieties grown in different regions require distinct photoperiod sensitivity^33^. In the OX*OsAFB6* mutant, grain yield was greatly increased by extending the vegetative growth phase due to delayed heading (Table 1) and an increased CK concentration in young panicles (Fig. 4). Plants with suitable overexpressed levels of *OsAFB6* also exhibited late flowering, more primary branches and more grain yield (Table 2). Hence, it is encouraged to generate several overexpression transgenic lines with different expression levels of *OsAFB6*. Then, all transgenic lines should be recorded for heading date and grain yield in regions with diverse photoperiod and temperature resources. Finally, the most appropriate lines for a region should be determined with an optimal heading date, which will allow the plants to fully utilize the local sunlight and temperature resources to produce high yield potential.

## Materials and Methods

### Generation of transgenic plants

A 1246-bp genomic fragment of *CCT05* from Minghui63 (MH63) and a 1812-bp coding sequence region of *OsAFB6* from Zhonghua 11 (ZH11) were separately cloned into the pCAMBIA1301 vector under the 35S promoter. For the *OsAFB6*-RNAi construct, a 472-bp fragment was amplified from ZH11 and cloned into the pDS1301 vector. For the *OsAFB6*-CRISPR construct, the target site was located in the first exon. For promoter-GUS assays, a 2-kb promoter fragment of *OsAFB6* was cloned into the DX2181 vector. Constructs were transformed into ZH11, a japonica rice variety (*O. sativa L. ssp. japonica*). For each transformation, we generated transgenic-negative plants from calli that did not integrate any exogenous DNA. They experienced the same tissue culture processes and were used as controls (hereafter named control plants) by Agrobacterium tumefaciens-mediated transformation methods^52^. The primer sequences for vector construction are listed in Table S2.

### Field experiments and trait measurements

In the normal rice growing season, rice plants were grown in the experimental field of Huazhong Agricultural University, Wuhan, China. Each year, seeds were planted in seedling beds in mid-May and were transplanted to the field approximately 25 days later. Ten plants were transplanted in each row as 16.5 × 26 cm within and between rows. Field management followed normal agricultural practices. Heading date (HD) was recorded as the number of days from sowing to the appearance of the first panicle. After ripening, the middle eight of ten plants in a row (the middle five out of seven plants for day length treatment) were measured for plant height (PH) from the paddy field surface to the highest panicle tip. At the harvest stage, the plants were harvested individually to score the number of panicles, the primary and secondary branch number (PBN; SBN), and the lengths of five panicles for each plant. Then, the numbers of full-filled grains and empty grains, as well as the full-filled grain length, width and weight, were obtained. Finally, spikelet number per panicle (SPP), 1000-grain weight (KGW) and grain yield per plant were calculated.

### Long-day (LD) and short-day (SD) condition treatments

In the field, the *OsAFB6* overexpression mutant (hereafter named OX*OsAFB6*) and its control plants were grown under natural long-day conditions (LD) in Wuhan (more than 13.5 hours of day length) and natural short-day conditions (SD) in Hainan (less than 12 hours of day length) for recording heading date and yield-related traits. OXOsAFB6 and its control plants were planted in nutrient solution (Table S3) under LD (daytime from 6:00 AM to 8:00 PM) and SD (daytime from 8:00 AM to 6:00 PM) conditions at 28 °C set in growth chambers. The leaves from three plants of each genotype at the same time point were pooled as a single biological sample, and three biological samples were used for checking the expression level of flowering genes by qRT-PCR.

### DNA extraction and Southern blotting

Leaves from seedlings were collected for DNA extraction following a previously described method^53^. A 150-bp fragment of the hygromycin gene was amplified by PCR to use as a probe and was labeled with DIG-High Prime from a DIG-High Prime DNA Labeling and Detection Starter Kit I (Roche, Mannheim, Germany). DNA concentrations were measured using a NanoDrop 2000 (Thermo, MA, USA), and 3 µg of DNA was digested with Hind III (Takara, Otsu, Japan) for Southern blotting.

### RNA extraction and qRT-PCR

Total RNA was isolated using TransZol reagent (TransGen Biotech, Beijing, China). Then, 3 μg of RNA was reverse-transcribed using oligo (dT) primers and MLV reverse transcriptase (Invitrogen, CA, USA) with the rice *Actin1* gene as a control for concentration consistency tests. The ABI7500 real-time PCR detection system was applied (Applied Biosystems, CA, USA). The rice *Ubq* gene (LOC_Os03g13170) was used as an internal control. Three technical repeats were performed for all analyses. All the primers used are listed in Table S2.

### Thermal asymmetric interlaced PCR (TAIL-PCR)

Three specific primers, SP1, SP2 and SP3, were designed according to the T-border sequence of the pCAMBIA vectors, along with six short arbitrary degenerate primers with low annealing temperatures (Table S2). The products from tertiary PCR with clear bands were digested using exonuclease. PCR systems and procedures were performed as previously described^54^.

### Bioinformatic analysis of *OsAFB6*

We searched the Rice Functional Genomic Express Database (http://signal.salk.edu/cgi-bin/RiceGE) to understand the gene structure. We used the conserved domain database on NCBI for conserved domain prediction, (https://www.ncbi.nlm.nih.gov/Structure/cdd/wrpsb.cgi). Promoter analysis was performed on PlantCARE (http://bioinformatics.psb.ugent.be/webtools/plantcare/html/). The expression pattern of *OsAFB6* was obtained from the Collection of Rice Expression Profiles on NCPGR (http://crep.ncpgr.cn/crep-cgi/home.pl) through a rice multiplatform microarray search^55^.

### Scanning electron microscopy observation of young panicles

Young panicles at the primary and secondary branch initiation stages were observed using a scanning electron microscope (JSM-6390LV, JEOL, Akishima-shi, Japan), as described previously^56^.

### RNA sequencing and qRT-PCR analysis

Young panicles (<1 mm) were dissected from the main culms of 80 OX*OsAFB6* mutant plants and 80 negative control plants in the summer of 2015 and then immediately placed into a 1.5 ml RNase-free tube (Invitrogen, CA, USA) floating in liquid nitrogen. Samples for each group were divided into two halves as 2 biological replicates for RNA extraction. RNA was extracted and sequenced by a biotechnology company (Novogene, Beijing, China). The RNA sequencing data were deposited in The National Center for Biotechnology Information Gene Expression Omnibus (NCBI GEO) database^57^ under the accession number GSE106954. Several key differentially expressed genes identified from RNA sequencing were later examined by qRT-PCR with RNA samples from young panicles (<1 mm) of 30 OX*OsAFB6* mutant plants and 30 negative control plants in the summer of 2016.

### Measurement of cytokinin and IAA concentrations

Young panicles (<1 mm) of 40 OX*OsAFB6* mutant plants and 40 control plants were dissected in the summer of 2017. Samples for each group were divided into 3 biological replicates. Samples in liquid nitrogen were delivered to Greensword Creation Technology Company (Wuhan, China). Measurements of IAA and four active forms of cytokinin were made using the methods described by Liu *et al*.^58^ and Chen *et al*.^59^. Three technical repeats were performed for all measurements.

## Electronic supplementary material

Dataset 1

Dataset 2

Dataset 3

## Data Availability

The data were deposited into the NCBI GEO database under the accession number GSE106954.
