# Supplementary material for: Overexpression of an auxin receptor OsAFB6 significantly enhanced grain yield by increasing cytokinin and decreasing auxin concentrations in rice panicle
Source: Sci Rep. 2018 Sep 19;8:14051. doi: 10.1038/s41598-018-32450-x (PMC6145926; doi:10.1038/s41598-018-32450-x)
Supplement: Supplementary file 1 — Dataset 1 [file 41598_2018_32450_MOESM1_ESM.pdf]

Supplementary information

Overexpression of an auxin receptor *OsAFB6* significantly enhanced grain yield by increasing cytokinin and decreasing auxin concentrations in rice panicle

Qin He, Lin Yang, Wei Hu, Jia Zhang, Yongzhong Xing

National Key Laboratory of Crop Genetic Improvement and National Center of Plant Gene Research (Wuhan), Huazhong Agricultural University, China

* yzxing@mail.hzau.edu.cn

Legends

Table S1. Cis-elements predicted in the promoter of *OsAFB6*.

Table S2. Primers used for all the experiments.

Table S3. Recipe for the nutrient solution (1000×).

Eight reagents stored separately, 50ml of each were added into 50L water in a box.

Figure S1. *CCT05* expression in the mutant and control plant.

(A) long-day condition. **(B)** short-day condition. OX*OsAFB6* and Control plant represent the *OsAFB6* over expression mutant and the negative Zhonghua 11 plant as control, respectively. The white bars indicate the light period, and the black bars indicate dark period. The numbers below the x-axis indicate hours of the days. Error bars, standard deviation.

Figure S2. Copy number analysis and expression of neighboring genes around the insertion site in the mutant.

(A) Southern blot of three individual mutants. **(B)** Inserted site shown by the red arrow and the positions of genes nearby. **(C, D, E)** Expression levels of LOC_Os03g08860, LOC_Os03g08870 and LOC_Os03g08850 for every 3 hours in a day under LD. **(G)** Expression level of LOC_Os03g08850 under SD. **(F, H)** expression levels of LOC_Os03g08850 in the control plant under LD and SD conditions. OX*OsAFB6* and Control plant represent the *OsAFB6* over expression mutant and the negative Zhonghua 11 plant as control, respectively. The white bars indicate the light period, and the black bars indicate dark period. The numbers below

the x-axis indicate hours of the days. Error bars, standard deviation.

Figure S3. Phenotype and expression levels of segregated population compared with OX*OsAFB6* and the control plants.

(A) Phenotype of early flowering and late flowering plants. **(B)** Phenotype of the control plant and the *OsAFB6* over expression mutant. **(C)** Their expression levels of *OsAFB6*. EF plant: early flowering plant; LF plant: late flowering plant. The early and late flowering plants were from the segregated F_2 population from the cross between the *OsAFB6* over expression mutant and control plant. ** indicated the significance level of $P < 0.01$.

Figure S4. Phenotype of double strains RNA interfered *OsAFB6* knockdown and CRIPSR *OsAFB6* knockout plants compared with the corresponding negative plants as control.

(A) Phenotype of control (left) and positive (right) double strains RNA interfered *OsAFB6* knockdown transgenic plants. **(B)** Phenotype of control (left) and positive (right) CRIPSR *OsAFB6* knockout plants.

Figure S5. *OsAFB6* gene structure with conserved domain predicted by BLAST.

The white bars indicate the 5' and 3' untranslated regions; the black bars indicate exon regions; the blue bar represents the F-box domain, as a recognizer for ubiquitination targets, located in the first exon; the red bars represent two AMN1 domains, containing LRR repeats. Both AMN1 domains were separated by intron regions.

Figure S6. Expression levels of flowering genes in the mutant and control plant under long day and short day condition.

OX*OsAFB6* and Control plant represent the *OsAFB6* over expression mutant and the negative Zhonghua 11 plant as control, respectively. The white bars indicate the light period, and the black bars indicate dark period. The numbers below the x-axis indicate hours of the days. Error bars, standard deviation.

Figure S7. Differential expressed genes according to RNA-seq.

In the OX*OsAFB6* mutant, there were 58 genes up regulated (red dots) and 229 genes down regulated (green dots) compared to the control plant.

Table S1. Cis-elements predicted in the promoter of *OsAFB6*.

Site Name	Sequence	Function	Frequency
5UTR Py-rich stretch	tttcttctct	cis-acting element conferring high transcription levels	1
TATA-box	tttta	core promoter element around -30 of transcription start	24
CAAT-box	caaat	common cis-acting element in promoter and enhancer regions	24
ACE	acgtgga	cis-acting element involved in light responsiveness	1
G-Box	cacgtt	cis-acting regulatory element involved in light responsiveness	4
ATCT-motif	aatctaatct	part of a conserved DNA module involved in light responsiveness	1
Box I	tttcaaa	light responsive element	1
GT1-motif	ggttaa	light responsive element	1
Sp1	cc(g/a)ccc	light responsive element	22
CATT-motif	gcattc	part of a light responsive element	1
GAG-motif	agagatg	part of a light responsive element	1
GATA-motif	gatagga	part of a light responsive element	1
AuxRR-core	ggtccat	cis-acting regulatory element involved in auxin responsiveness	1
ABRE	tacgtg	cis-acting element involved in the abscisic acid responsiveness	1
TATC-box	tatccca	cis-acting element involved in gibberellin-responsiveness	1
HSE	aaaaaatttc	cis-acting element involved in heat stress responsiveness	1
TC-rich repeats	attttcttca	cis-acting element involved in defense and stress responsiveness	1
GC-motif	cccccg	enhancer-like element involved in anoxic specific inducibility	3
O2-site	gatgatgtgg	cis-acting regulatory element involved in zein metabolism regulation	1
Skn-1_motif	gtcat	cis-acting regulatory element required for endosperm expression	1
GCN4_motif	caagcca	cis-regulatory element involved in endosperm expression	1

AAGAA-motif	gaaagaa	3
TATCCAT/C-motif	tatccat	1
CTAG-motif	actagcagaa	1
AC-I	cccacctacc	2
AC-II	ccaccaaccccc	1

Table S2. Primers used for all the experiments.

Gene	PCR	Forward primer (F) 5'- 3'	Reverse primer (R) 5'- 3'
hygromycin	Probe for southern	cgaagcccgctgctgcga	tccttgccgagctgggat
SP1	primary reaction from left border	gaagtactcgccgatagtggaaacc	
SP2	secondary reaction from left border	atagggtttcgctcatgtgttgagcat	
SP3	tertiary reaction from left border	ccagtactaaaatccagatcccccgaat	
NTLB5	sequencing from left border	aatccagatcccccgaatta	
PFRB4	sequencing from right border	tgcaggttctctccaaatga	
AD2-1	AD primer	(agct)gacga(gc)(at)ga(agct)a(at)gaa	
AD2a	AD primer	(agct)gtcga(gc)(at)ga(agct)a(at)gaa	
AD8	AD primer	ag(at)g(agct)ag(at)a(agct)ca(at)agg	
AD10	AD primer	(at)gtg(agtc)ag(at)a(agct)ca(agct)aga	
AD11	AD primer	tg(at)g(agct)ag(gc)a(agct)ca(gc)aga	
OsAFB6	Subcellular localization	gaattcatgtccgaggaggacgacga	gaattctaggatcttcacgaatggtg
Ghd7	Subcellular localization	gaattcatggggatggccaatgagga	gaattctctgaaccattgtccaagct
CCT05	qRT	ctaccttctagtgttctagctcatc	caactagaactttggctaatcctatg
CCT05	OX	cccgggatggagatggagctagggtt	ggatccctagaaggtaggcacga
LOC_Os03g08850	qRT	ctccggaatgcaccatttcta	cgttctcaggctggctattt
LOC_Os03g08860	qRT	ccttcaacgagcagaacaaatg	ctttacggggctgtcatagt
LOC_Os03g08870	qRT	ctgcaaatgactctgacaaagg	gacgagagcgaagaggattac
Ehd1	qRT	tggaaatctcgaaaaacccg	gcgctagcaaagcttcggt
Hd3a	qRT	gctcactatcatcatccagcatg	ccttgctcagctatttaattgcataa
RFT1	qRT	tgacctagattcaaagtctaatcctt	tgccggccatgtcaaattaataac
OsGI	qRT	atcgttctgcaggccgaga	tcaccaatgcttctgggctat
Ghd7	qRT	aggtgctacgagaagcaaatcc	gggcctcatctcggcatag
Ghd7.1	qRT	aatggaagcaatgggcagaatggg	tgatcactgcagccactctatgct
Hd1	qRT	tcagcaacagcatatctttctcatca	tctggaatttggcatatctatcacc

Ubq	qRT	aaccagctgaggcccaaga	acgattgatttaaccagtccatga
OsIAA1	qRT	ggtgaacgggacggagta	ctggcaagtttccacaaacatc
OsIAA2	qRT	ccgcctacttctcctcct	tggcttggaggcattgg
OsIAA3	qRT	acaaggatggtgactggatg	ccaattgcatctgagcctttc
OsIAA4	qRT	cccatgtcctcacctatgaag	cttgcaatcctgagcttctttac
OsIAA6	qRT	tggaagctgagagaagcattag	accaggaagaccaagctttag
OsIAA7	qRT	ccagcaaagcatctcttgaac	gggacaccatccatgtttatct
OsIAA8	qRT	tctgacatacgaagaccaggag	ccgctcttgcaatcctcaat
OsIAA9	qRT	ccaccaaggcgagaagaaa	cccaaccagcatccaatcc
OsIAA10	qRT	ggatgctcgttggagatgtt	atcgctgaccaagaccattc
OsIAA11	qRT	agttgtccatggcgttcc	ctctcagcttattgctctccttc
OsIAA12	qRT	ggtgaggtcgtacaggaaga	gtgatgggctcgtctttgtt
OsIAA13	qRT	ccagtcagtgaagagcaagaa	gaccttcacgaaggcagag
OsIAA14	qRT	ttcgccgtcgcctatga	cgcattatccgcagcttctt
OsIAA15	qRT	aaggccaagttcgtgaagg	gatggtgaagtgggagaagaac
OsIAA16	qRT	aggaagagaggagccttga	tttcccttggctccatcag
OsIAA17	qRT	gcaagaacacgatggctacta	ctgaccttgacgtacaggaac
OsIAA18	qRT	gagagcagctgattcaagagag	tggtaggctttgaatgtgtagg
OsIAA19	qRT	atcttccttgggacttgtttact	ttgttctgacctgtctgttcc
OsIAA20	qRT	acccatacgctgtcaccta	accagtatcttgagccgtttc
OsIAA21	qRT	ctctttggctctggagaagatg	catcaggcggcaatcagata
OsIAA22	qRT	tcagatggcattgcaactaga	catgcggtctccctcattatc
OsIAA23	qRT	gtcaaggtggccgtgga	gcgaacatgccgtggag
OsIAA24	qRT	cctcttcaccaagtgcttctc	cagggcacatctccaacaa
OsIAA25	qRT	tcagatggcattgcaactagag	catgcggtctccctcattatc
OsIAA26	qRT	gtacttcgtgaaggtgagcat	agcagtagaacatgtcgttgag
OsIAA27	qRT	tgcacatggatgggtacaag	atgagtcatcttggtgaggac
OsIAA28	qRT	gaatatcaggaggaggaagagga	gaccgaccaaagatggtttct
OsIAA29	qRT	ctgctgacgaagatggagatg	ggtcatcgttgctcgtgtat
OsIAA30	qRT	attggaaactgtgggtctcat	cctcgtaggttggcacatatt
OsIAA31	qRT	gcagcagaaggaggatgtc	gtagcccttgtacaccttgag
AUX1	qRT	ctgatcgcctgtgcaagtaa	cagatgcggtagttgtggaa
PIN1	qRT	gtctgcttcaggtggaactt	gaacagaccgagactgaacat
PIN2	qRT	cgtctccttcaggtggaatatc	catgaacaagcctaagctgaac
PIN3	qRT	gtcgagaagtccatctccattc	ttgccacacgcgatgat
PIN5a	qRT	ccctacctcaatccatcacatc	gtagggagacaagcattccaa
OsCKX2	qRT	atctaccccatgaaccgcaac	tgcacgaatcttggccaga

OsAFB6	OX	cccgggatgtccgaggaggacgacga	ggatccttataggatcttcacgaatg
OsAFB6	RNAi	actagtggtaccttcgctggagacagca atct	gagctcggatcctgcagctctgata gatggct
OsAFB6	CRISPR	gtgtaccggtcccgcgccgagtcg	aaaccgactcggcgcgggaccggt
OsAFB6	Promoter-GUS	ggatccgactgcaagcaccgaatgct	ctgcaggaggaactggagcaccgt

Table S3. Recipe for the nutrient solution (1000×).

	reagent	g/L
1	NH_4NO_3	114.3g
2	$NaH_2PO_4 \cdot 2H_2O$	50.4g
3	K_2SO_4	89.3g
4	$CaCl_2$	110.8g
5	$MgSO_4 \cdot 7H_2O$	405g
6	$NaSiO_3 \cdot 9H_2O$	47.6g
	$EDTA \cdot Na_2 \cdot 2H_2O$	18.85g/500ml
7	$FeSO_4 \cdot 7H_2O$	13.98g/500ml
	Water bath at 65℃, then mix the two reagents after cool to room	
	$MnCl_2 \cdot 4H_2O$	1.875g
	$(NH_4)_6Mo_7O_{24}$	0.0925g
	H_3BO_3	1.1675g
	$ZnSO_4 \cdot 7H_2O$	0.04375g
8	$CuSO_4 \cdot 5H_2O$	0.03875g
	$FeCl_3 \cdot 6H_2O$	9.625g
	Citric acid$\cdot H_2O$	14.875g
	70% H_2SO_4	62.5ml

Eight reagents stored separately, 50ml of each were added into 50L water in a box.

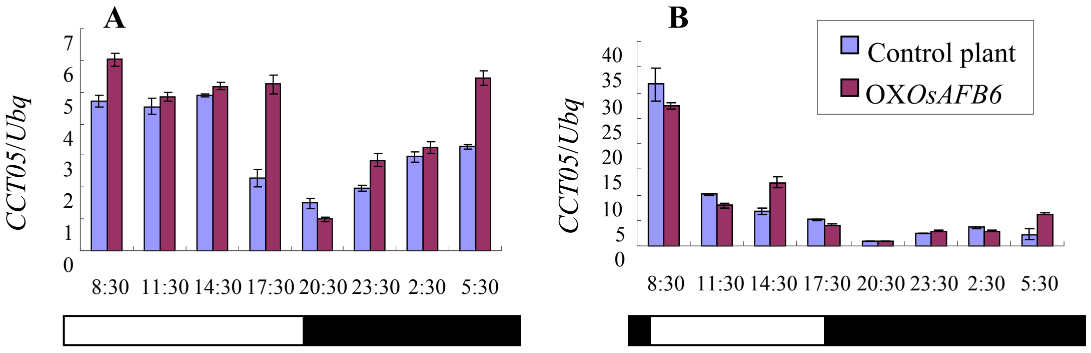

Figure S1. *CCT05* expression in the mutant and control plant.

(A) long-day condition. **(B)** short-day condition. OX*OsAFB6* and Control plant represent the *OsAFB6* over expression mutant and the negative Zhonghua 11 plant as control, respectively. The white bars indicate the light period, and the black bars indicate dark period. The numbers below the x-axis indicate hours of the days. Error bars, standard deviation.

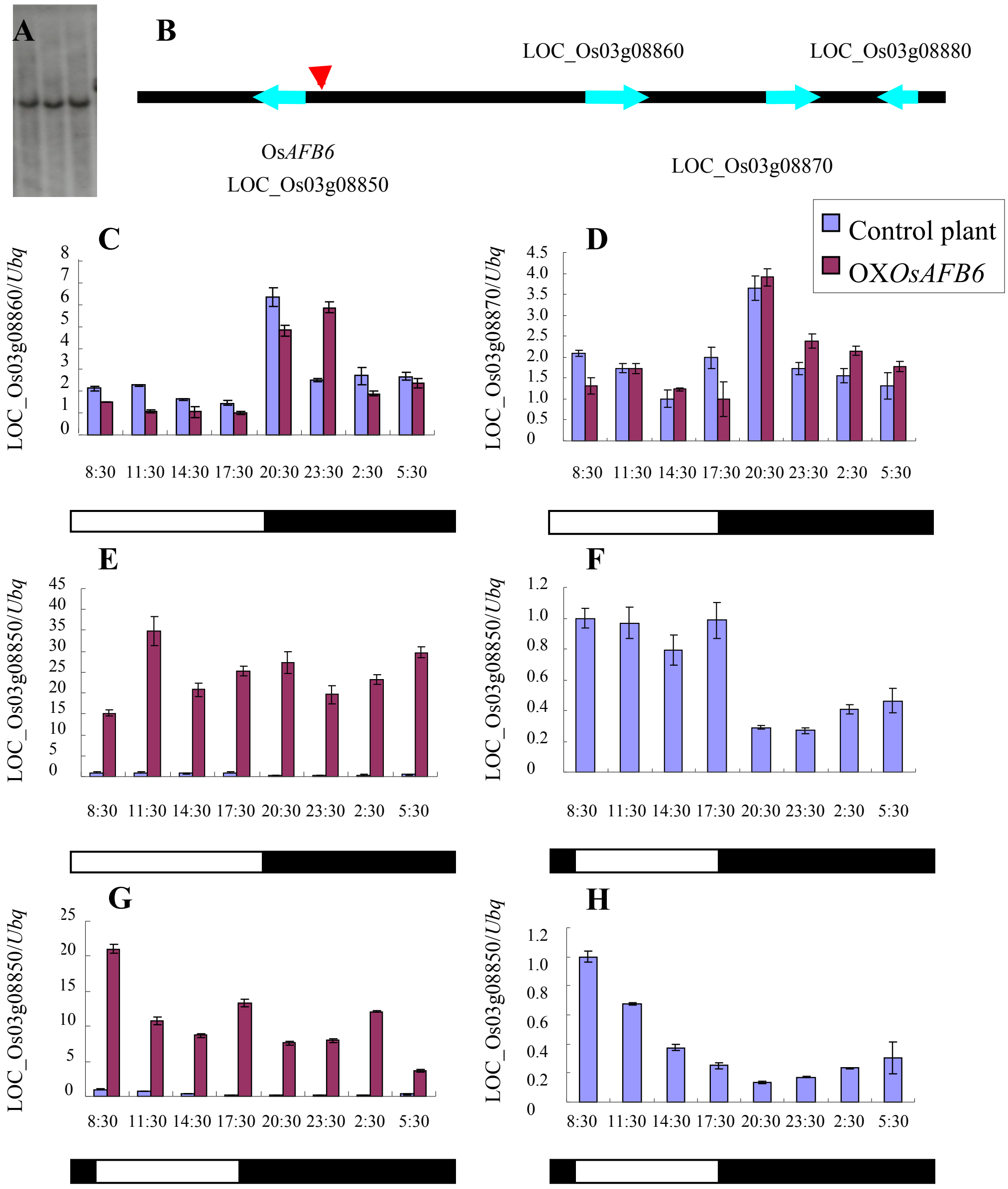

Figure S2. Copy number analysis and expression of neighboring genes around the insertion site in the mutant.

(A) Southern blot of three individual mutants. **(B)** Inserted site shown by the red arrow and the positions of genes nearby. **(C, D, E)** Expression levels of LOC_Os03g08860, LOC_Os03g08870 and LOC_Os03g08850 for every 3 hours in a day under LD. **(G)** Expression level of LOC_Os03g08850 under SD. **(F, H)** expression levels of LOC_Os03g08850 in the control plant under LD and SD conditions. OX*OsAFB6* and Control plant represent the *OsAFB6* over expression

mutant and the negative Zhonghua 11 plant as control, respectively. The white bars indicate the light period, and the black bars indicate dark period. The numbers below the x-axis indicate hours of the days. Error bars, standard deviation.

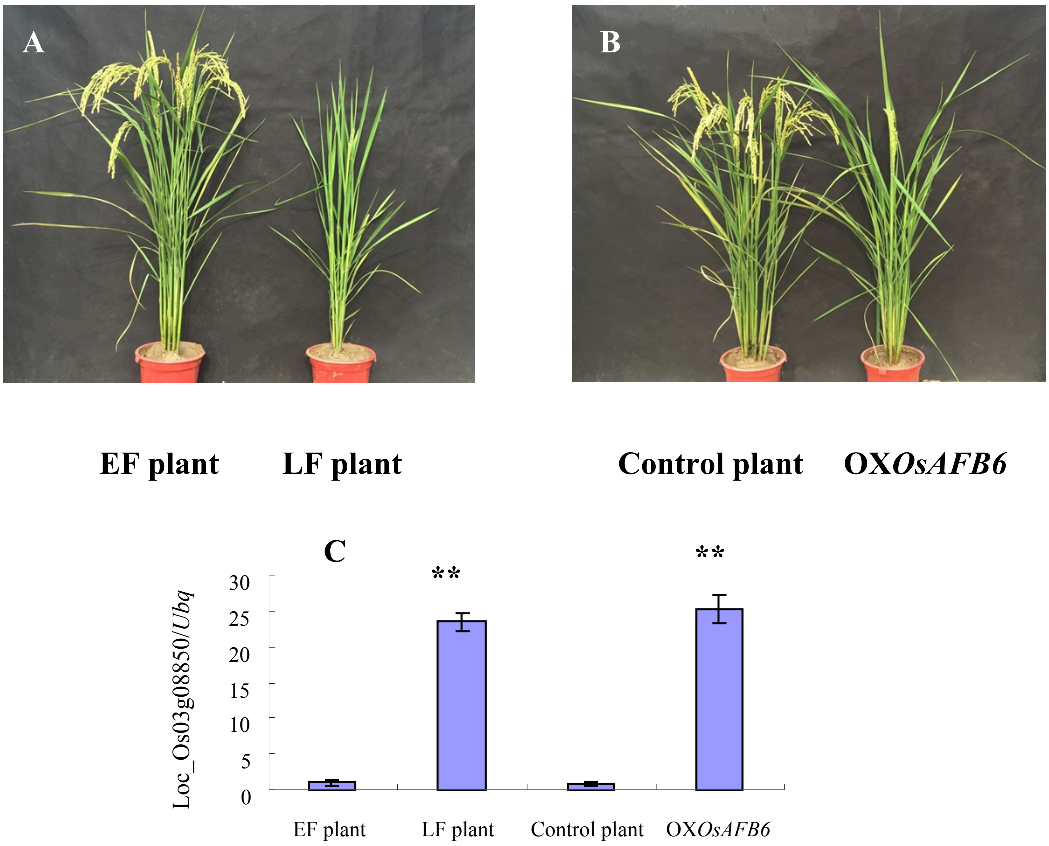

Figure S3. Phenotype and expression levels of segregated population compared with OX*OsAFB6* and the control plants.

(A) Phenotype of early flowering and late flowering plants. **(B)** Phenotype of the control plant and the *OsAFB6* over expression mutant. **(C)** Their expression levels of *OsAFB6*. EF plant: early flowering plant; LF plant: late flowering plant. The early and late flowering plants were from the segregated F_2 population from the cross between the *OsAFB6* over expression mutant and control plant. ** indicated the significance level of $P < 0.01$.

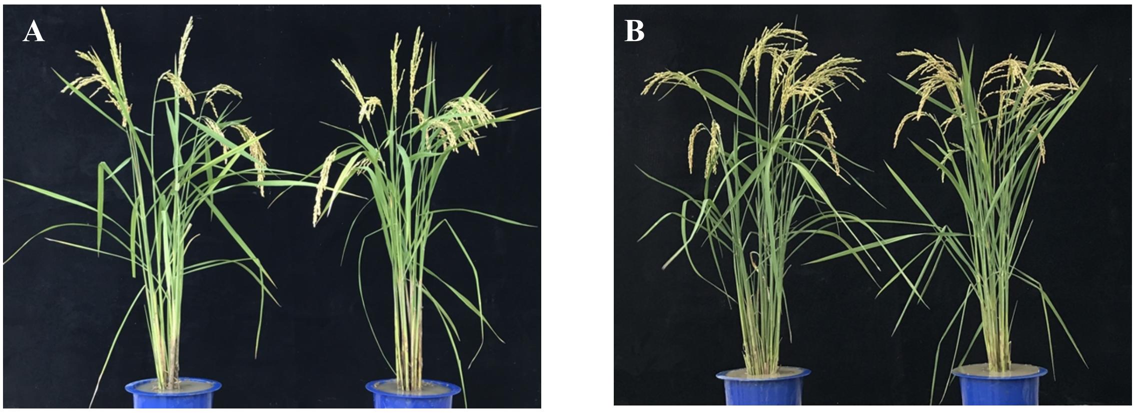

RNAi*OsAFB6*(-) RNAi*OsAFB6*(+) CRISPR*OsAFB6*(-) CRISPR*OsAFB6*(+)

Figure S4. Phenotype of double strains RNA interfered *OsAFB6* knockdown and CRIPSR *OsAFB6* knockout plants compared with the corresponding negative plants as control.

(A) Phenotype of control (left) and positive (right) double strains RNA interfered *OsAFB6* knockdown transgenic plants. **(B)** Phenotype of control (left) and positive (right) CRIPSR *OsAFB6* knockout plants.

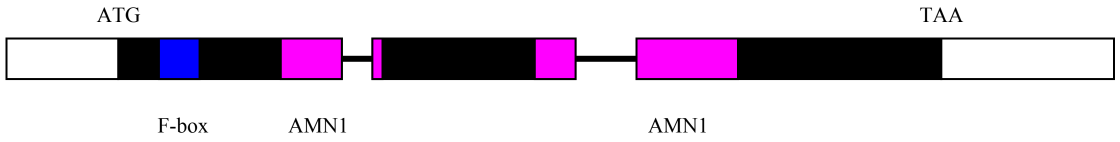

Figure S5. *OsAFB6* gene structure with conserved domain predicted by BLAST.

The white bars indicate the 5' and 3' untranslated regions; the black bars indicate exon regions; the blue bar represents the F-box domain, as a recognizer for ubiquitination targets, located in the first exon; the red bars represent two AMN1 domains, containing LRR repeats. Both AMN1 domains were separated by intron regions.

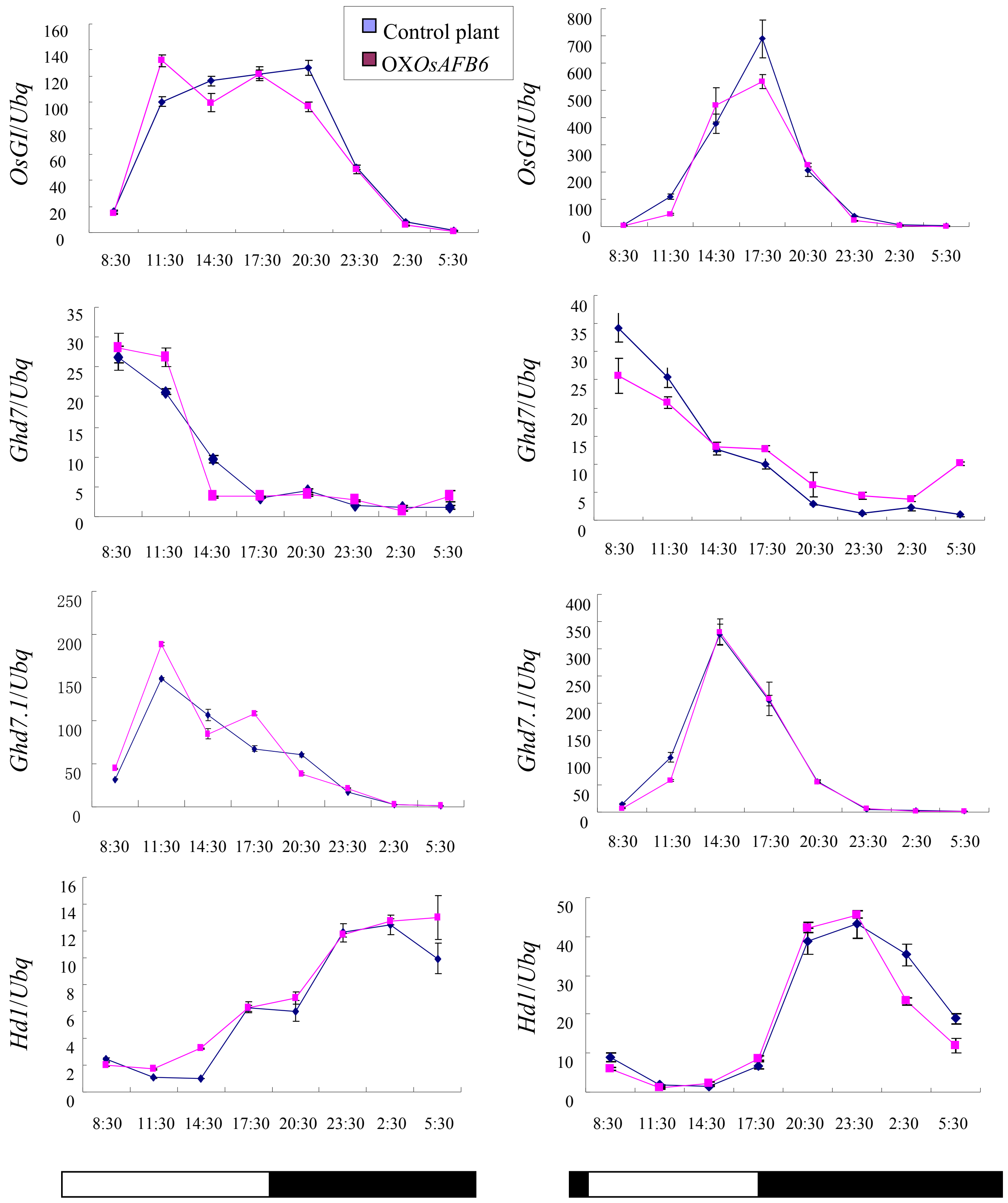

Figure S6. Expression levels of flowering genes in the mutant and control plant under long day and short day condition.

OX*OsAFB6* and Control plant represent the *OsAFB6* over expression mutant and the

negative Zhonghua 11 plant as control, respectively. The white bars indicate the light period, and the black bars indicate dark period. The numbers below the x-axis indicate hours of the days. Error bars, standard deviation.

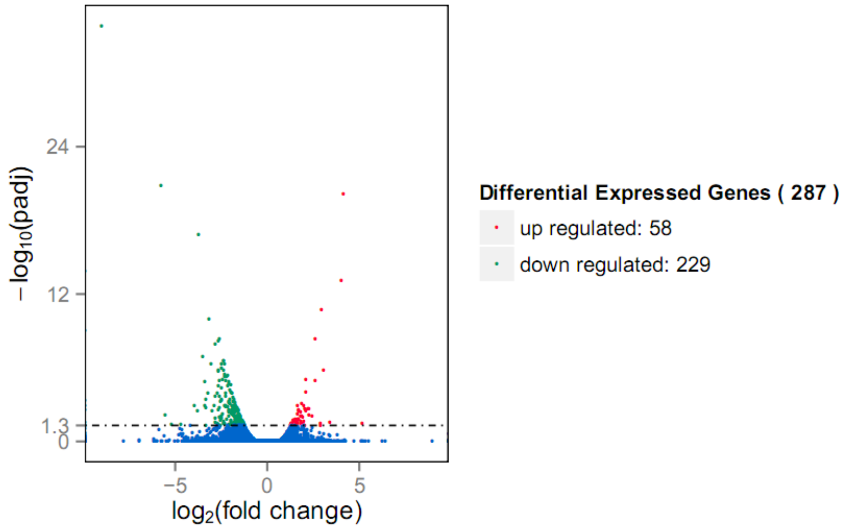

Figure S7. Differential expressed genes according to RNA-seq.

In the OX*OsAFB6* mutant, there were 58 genes up regulated (red dots) and 229 genes down regulated (green dots) compared to the control plant.
