# Supplementary material for: Overexpression of an auxin receptor OsAFB6 significantly enhanced grain yield by increasing cytokinin and decreasing auxin concentrations in rice panicle
Source: Sci Rep. 2018 Sep 19;8:14051. doi: 10.1038/s41598-018-32450-x (PMC6145926; doi:10.1038/s41598-018-32450-x)
Supplement: Supplementary file 3 — Dataset 3 [file 41598_2018_32450_MOESM3_ESM.pdf]

Supplementary information

Overexpression of an auxin receptor *OsAFB6* significantly enhanced grain yield by increasing cytokinin and decreasing auxin concentrations in rice panicle

Qin He, Lin Yang, Wei Hu, Jia Zhang, Yongzhong Xing

National Key Laboratory of Crop Genetic Improvement and National Center of Plant Gene Research (Wuhan), Huazhong Agricultural University, China

* yzxing@mail.hzau.edu.cn

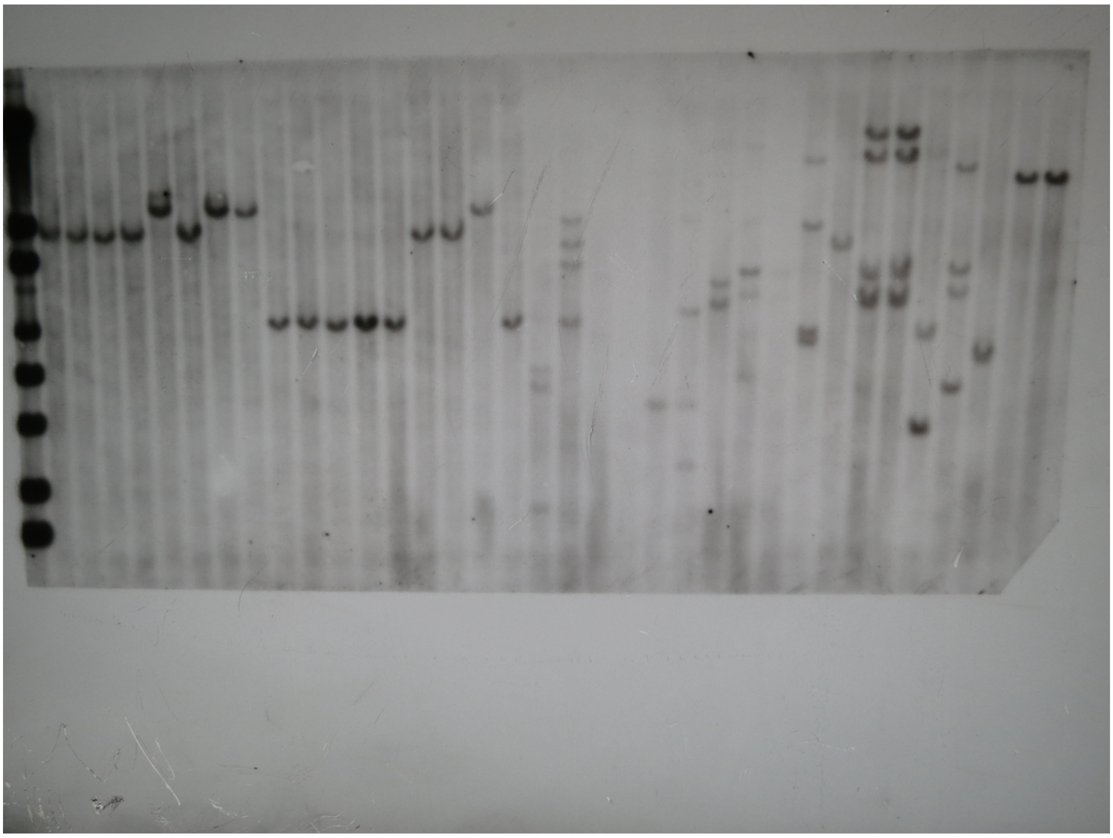

original picture of Southern Blot result,we cropped lane 2-4 in Fig S2
